# Molecular Basis of Differential Selectivity of Cyclobutyl-Substituted Imidazole Inhibitors against CDKs: Insights for Rational Drug Design

**DOI:** 10.1371/journal.pone.0073836

**Published:** 2013-09-13

**Authors:** Soumya Lipsa Rath, Sanjib Senapati

**Affiliations:** Department of Biotechnology, Indian Institute of Technology Madras, Chennai, India; Oak Ridge National Laboratory, United States of America

## Abstract

Cyclin-dependent kinases (CDKs) belong to the CMGC subfamily of protein kinases and play crucial roles in eukaryotic cell division cycle. At least seven different CDKs have been reported to be implicated in the cell cycle regulation in vertebrates. These CDKs are highly homologous and contain a conserved catalytic core. This makes the design of inhibitors specific for a particular CDK difficult. There is, however, growing need for CDK5 specific inhibitors to treat various neurodegenerative diseases. Recently, cis-substituted cyclobutyl-4-aminoimidazole inhibitors have been identified as potent CDK5 inhibitors that gave up to 30-fold selectivity over CDK2. Available IC_50_ values also indicate a higher potency of this class of inhibitors over commercially available drugs, such as roscovitine. To understand the molecular basis of higher potency and selectivity of these inhibitors, here, we present molecular dynamics simulation results of CDK5/p25 and CDK2/CyclinE complexed with a series of cyclobutyl-substituted imidazole inhibitors and roscovitine. The atomic details of the stereospecificity and selectivity of these inhibitors are obtained from energetics and binding characteristics to the CDK binding pocket. The study not only complements the experimental findings, but also provides a wealth of detailed information that could help the structure-based drug designing processes.

## Introduction

Cyclin-dependent kinases (CDKs) play crucial roles in eukaryotic cell division cycle. They belong to the CMGC subfamily of protein kinases and assist the γ-phosphate transfer from ATP to peptide substrates [1], [2]. At least seven different CDKs have been reported to be implicated in the cell cycle regulation in vertebrates. Among these, CDK2 functions during the progression of cell cycle from the G1 to S phase [3], [4]. CDK2, like most of the other CDKs, follows a two-step process to become fully functional: (i) the association with the regulatory subunit – cyclin A or cyclin E, (ii) phosphorylation of residue Thr160 located in the so-called activation loop [5], [6]. However, certain CDKs, *e.g.* CDK5 do not follow this mode of activation. The activity of CDK5 is restricted to nervous system by the localization of its activators p25/p35/p39, the binding of which makes CDK5 fully active without the subsequent requirement of phosphorylation of the activation loop residue [7], [8]. While aberrant activity of CDK2 has been identified in a number of diseases including cancer, embryonic lethality, male sterility etc., the deregulation of CDK5 causes serious neurodegenerative disorders, e.g. Alzheimer’s disease, lateral sclerosis, stroke etc [9]–[11].

**Figure 1:**
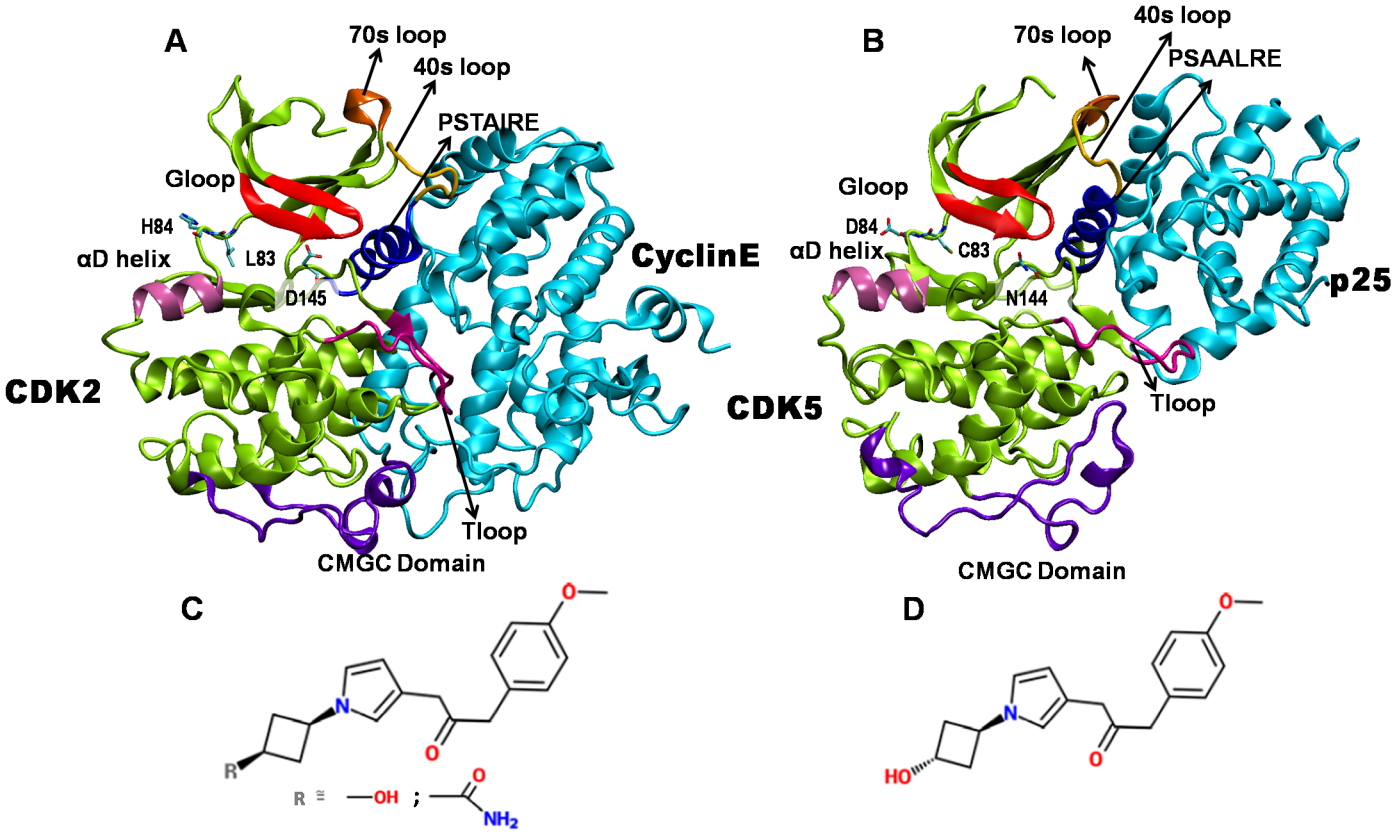
Structures of active CDKs and imidazole inhibitors. (A) CDK2/cyclinE complex, (B) CDK5/p25 complex, (C) cis-OH or cis-N-acetyl inhibitor, and (D) trans-OH inhibitor. In (A) and (B), CDKs are shown in green and the activators are shown in cyan. The functionally relevant regions of CDKs are highlighted: G-loop (red), PSTAIRE/PSAALRE helix (magenta), T-loop (blue), α-D helix (pink), 40s (yellow), 70s loop (orange), and CMGC conserved kinase domain (purple). The CDK2/CDK5 variant residues in substrate binding pocket are shown in licorice.

CDKs are highly homologous and contain a conserved catalytic core. For example, CDK2 and CDK5 share a sequence homology of 60%, with the substrate binding pocket alone showing nearly 93% sequence similarity [8], [12]. The 3D structures of CDKs are mainly composed of two domains, the N and the C-terminal domains (Figure 1) [13], [14]. The catalytic cleft that binds ATP is located at the interface of these two domains. A glycine rich loop, commonly known as G-loop, lies above the ATP binding pocket and is conserved in many kinases. The primary function of this loop is to align the substrate and ATP correctly, for a smooth transfer of the γ-phosphate [15]–[17]. The N-terminal domain is primarily composed of a β-sheet, containing five antiparallel β-strands, and one α-helix. This helix with the “PSxAxRE” motif is a signature of this class of proteins and constitutes the main point of interaction with activator proteins. The loop which precedes the PSxAxRE helix, known as the 40s loop, also interacts with the activator protein. The C-terminal domain is predominantly α-helical and contains the so-called T-loop, the residue Thr160 of which becomes phosphorylated by CAK for CDK2 activation [13]–[18]. However, CAK does not phosphorylate CDK5 on the analogous Ser159 [8], [18]. The catalytic pockets of CDK2 and CDK5 are primarily comprised of 20 residues, three of which differ from CDK2 to CDK5 as follows: Lys83 to Cys83, His84 to Asp84 and Asp145 to Asn144 [12]. The respective partner proteins, Cyclin E and p25, though have less sequence homology, are structurally similar with both possessing the typical cyclin box fold.

Due to their key regulatory roles, CDKs have become important pharmaceutical targets for inhibitor design [9], [19]. There is a particular demand for CDK5 specific inhibitors to treat various neurodegenerative diseases [20]. However, it is difficult to design the inhibitor specific to a particular CDK due to the structural homology among number of CDKs [4]. Very recently, Helal et al. have identified novel cis-substituted cyclobutyl-4-aminoimidazole inhibitors that gave improved enzyme and cellular potency against CDK5/p25 with up to 30-fold selectivity over CDK2/Cyclin E [21]. To understand the molecular basis of higher potency of these inhibitors, here we carry out all-atom molecular dynamics simulations of active CDK5/p25 and CDK2/CyclinE bound to a series of cyclobutyl-substituted imidazole inhibitors. The atomic details of the stereospecificity and selectivity of these inhibitors are obtained from energetics and binding characteristics to the CDKs.

## Materials and Methods

### Simulation Details

The initial structures of inhibitor-bound CDK2/Cyclin E and CDK5/p25 complexes were obtained by docking the inhibitors in the available crystal structures of active CDK2 (PDB ID: 1W98) and CDK5 (PDB ID: 3O0G) [22], [23]. We considered three different imidazole inhibitors in this study: N-[1-(cis-3-hydroxycyclobutyl)-1H-imidazol- 4-yl]-2-(4-methoxyphenyl)acetamide, N-[1-(trans-3-hydroxy cyclobutyl)-1H-imidazol-4-yl]-2-(4-methoxyphenyl)acetamide, and N-{1-[cis-3-(acetylamino)cyclobutyl]-1H-imidazol- 4-yl}-2-(4-methoxyphenyl)acetamide. Hereafter these molecules are abbreviated as cis-OH, trans-OH, and cis-N-acetyl, respectively, and their chemical structures are included in Fig. 1. *In vivo* and *in vitro* studies have shown distinctly different inhibitory effects of these molecules on CDK2 and CDK5 [21]. Table 1 lists the experimentally determined IC_50_ values of these inhibitors.

**Table 1:** Reported IC_50_ values of the selected inhibitors in nM.

Inhibitor	CDK2/CyclinE	CDK5/p25
cis-OH	66.5	93
trans-OH	763	1090
cis-N-acetyl	63	9
roscovitine	700	160

Data are collected from Refs. 21,42.

As the kinase inhibition assay was performed in active complexes, the CDK-inhibitor interactions were examined in presence of the activators, cyclin E and p25 for CDK2 and CDK5, respectively. For this purpose, the crystal structure coordinates of cis-OH and cis-N-acetyl were extracted from their bound complex with CDK2 (PDB ID: 3IGG and 3IG7, respectively, [21]) and were docked manually to CDK2/Cyclin E and CDK5/p25 complexes by superposing the CDK structures without changing the inhibitor coordinates. A similar docking protocol has been adopted earlier to study the protein-ligand interactions and was validated by comparing with the available crystal structures [24]–[26]. The corresponding trans-isomers were created and the structure were optimized by using Gaussian 03 program using B3LYP functional and 6–311+G* basis set, before docking to the CDKs [27]. The atom-centered RESP charges for all inhibitors were determined *via* fits to the electrostatic potentials obtained from the calculated wave functions. The missing interaction parameters in the inhibitors were generated using antechamber tools in Amber [28]. As controls, the crystal structures of roscovitine-bound active CDK2 and CDK5 complexes were also simulated (respective PDB IDs are: 3DDQ, 1UNL) [29], [30].

For simulations, the hydrogens for heavy atoms were added by leap program in Amber 11.0 package [28]. Added hydrogens were energy minimized for 1000 steps using the conjugate gradient and another 1000 steps using the steepest descent algorithm. The protonation states of histidines - HID or HIE - were determined by the local hydrogen bonding network using WHATIF program [31]. After relaxing the added atoms in gas phase, the structures were solvated in a cubic periodic box of explicit water with water molecules extending 9 Å outside the protein-complex on all sides. The 3-site TIP3P model was chosen to describe the water molecules [32]. To neutralize the systems, five Na^+^ ions for CDK2/CyclinE and one for CDK5/p25 were added. Subsequently, an extensive set of minimization and thermalization of the engineered structure was performed by maintaining harmonic restraints on the protein heavy atoms followed by gradually heating to 300K in a canonical ensemble. The harmonic restraints were gradually reduced to zero and solvent density was adjusted under isobaric and isothermal conditions at 1 atm and 300 K. The systems were equilibrated for 5 ns in NPT ensemble, with a simulation time step of 2 fs. During this period, the energy components, mass density, and RMSDs were seen to be converging. These structures were further simulated to generate the 50 ns production data. The two variants CDK2:L83C and CDK2:H84D were also simulated for 50 ns following the same protocol. For control roscovitine-bound CDK simulations, the production data was generated for 20 ns each. Thus a total of ten simulations were performed in the study (Table S1). The long-range electrostatic interactions were treated by using Particle-Mesh Ewald sum [33] and SHAKE was used to constrain all bonds involving hydrogen atoms. Amber11 molecular dynamics simulation package with Amber ff99SB force field was used for all simulations [34].

### Free Energy Calculations

Binding free energies (ΔG_bind_) of the inhibitors were calculated by Molecular Mechanics Poisson Boltzmann Surface Area (MMPBSA) approach [35]. For every system, the block averaged ΔG values were calculated from five independent windows of 2 ns (*i.e.* last 10 ns trajectory). The binding free energy of an inhibitor is obtained by taking the difference between the free energies of the protein-inhibitor complex (G_complex_), the unbound protein (G_receptor_), and the inhibitor (G_ligand_):
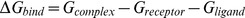
(1)


The ΔG_bind_ values were computed using the scripts available with AMBER 11 programme [28], where ΔG_bind_ is calculated from the changes in the molecular mechanical gas phase energy (ΔE_MM_), entropic contribution, and solvation free energy due to the binding of ligand to receptor for the formation of complex:
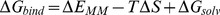


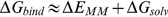
(2)


The MM gas phase energy term (ΔE_MM_) takes care of the electrostatic and van der Waal’s interactions between protein and ligand. The ΔG_solv_ is estimated by solving the linearised Poisson Boltzman equation for each of the three states (ΔG_polar_) and adding an empirical term for hydrophobic contributions to it (ΔG_nonpolar_). The hydrophobic contribution is calculated from the solvent accessible surface area. It is customary to neglect the entropic contribution (TΔS), as the calculations involve binding of similar type of ligands to the receptor. Hence, the computed values will be termed as the relative binding free energies. The experimental free energy of binding values (ΔG_expt_) was determined from the IC_50_ values by using the equation: ΔG = −RTlnIC_50_ [36], [37]_._

## Results and Discussion

### Binding of cis- and trans-OH to Active CDK2 and CDK5

To test the stability of the systems, we monitored the root mean squared deviations (RMSD) of the inhibitor-bound CDK complexes from the starting structures. The convergence of RMSD values at approximately 5 ns of the simulation time indicates that the systems were well equilibrated and have attained stability (Fig. S1). Interestingly, the cis-OH bound CDK complexes were found to exhibit significantly lower RMSDs than the corresponding trans complexes. RMSDs of the inhibitors alone in the complexes also show a similar trend (Fig. S2), implying a better binding of cis-OH inhibitor to both CDK2 and CDK5 binding pockets than the trans-OH inhibitor.

**Figure 2:**
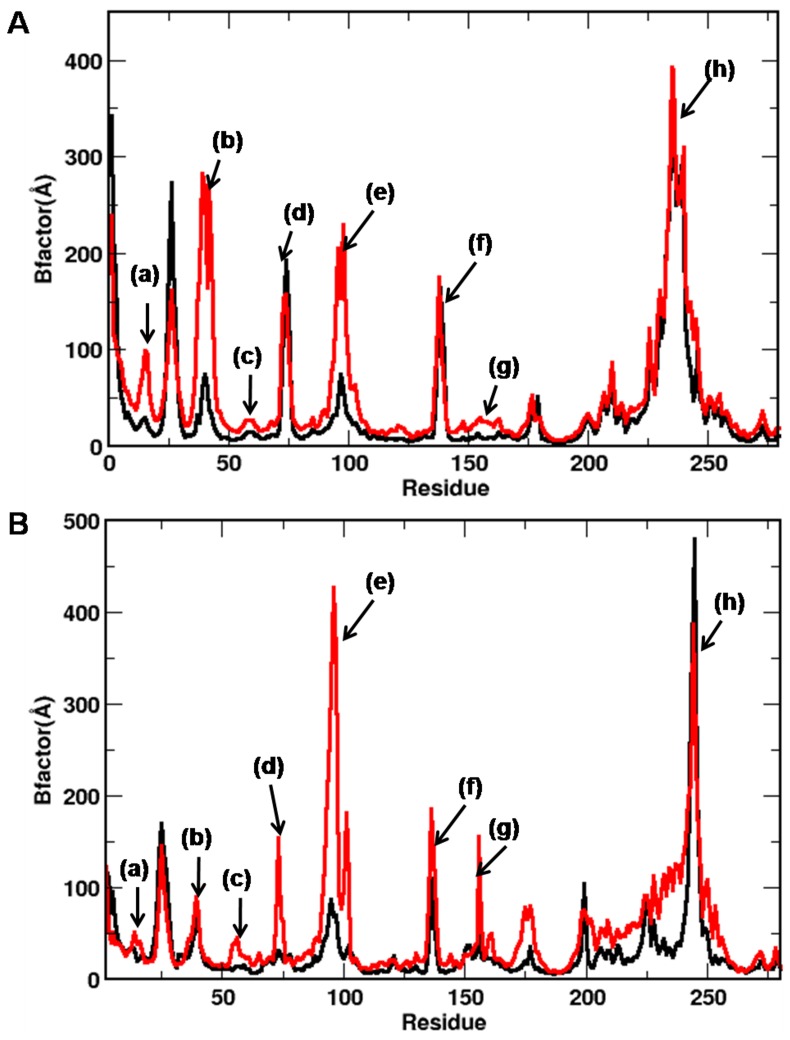
B-factors of CDKs bound with cis-OH (black) and trans-OH (red) inhibitors. Results are shown for (A) CDK2 and (B) CDK5 complexes. Highly fluctuating regions are labelled: (a) G-loop, (b) 40s loop (c) PSTAIRE helix, (d) 70s loop, (e) α-D helix, (f) substrate binding pocket, (g) T-loop, and (h) CMGC domain.

The analyses of local fluctuations of the CDK residues also suggest a stronger protein-inhibitor interaction in cis-OH, as exemplified by the lower B-factor values of the functionally relevant loops and helices (Fig. 2). For example, the G-loop and αD helix that are known to play crucial roles in ligand binding, show considerably reduced fluctuations in cis-OH-CDK complexes. Most of the other important regions of CDK, such as 40s loop, PSTAIRE helix, T-loop, and residues around substrate binding pocket also show reduced fluctuations in cis-OH-CDK2 complex. A similar trend was noticed for cis-OH-CDK5 complex. The modulated fluctuations of PSTAIRE/PSLAARE helix and 70s loop, which lie at CDK-cyclin/p25 interfaces, imply that the binding of inhibitors to substrate-binding pocket can also affect the binding of CDKs to the activators, allosterically [38]. Interestingly, all the inhibitor-bound complexes displayed high fluctuations around the conserved CMGC kinase domain.

**Figure 3:**
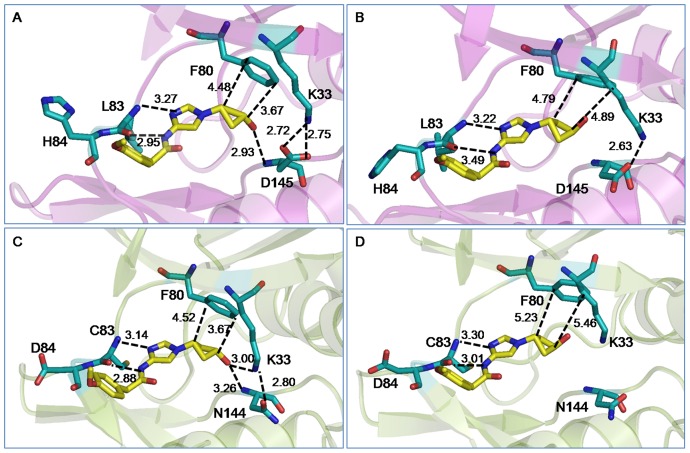
Average structures of the cis/trans-OH bound CDK complexes. For clarity, only the inhibitors and the adjacent protein residues are shown: (A) cis-OH bound CDK2, (B) trans-OH bound CDK2, (C) cis-OH bound CDK5, and (D) trans-OH bound CDK5. Possible modes of interactions are indicated by dotted lines with average distances shown. Color scheme: O: red; N: blue; protein C: cyan; inhibitor C: yellow. Hydrogens are omitted for clarity.

To obtain a better understanding of the interactions, we compared the average structures of the cis- and trans-OH bound CDK2 and CDK5 complexes. This is shown in Fig. 3. For clarity, only the inhibitor and the adjacent protein residues that involve in direct interactions are shown. Similar to the other ATP competitive inhibitors, both cis- and trans-OH inhibitors were found to interact effectively with the backbone of the protein. For example, the imidazole ring of the inhibitors involves in multiple interactions with hinge region residues Glu81, Phe82, Leu83/Cys83, and His84/Asp84 of CDK2/CDK5, mimicking the interactions of the ATP purine ring. The phenylacetamide group of the inhibitor was found to involve in hydrophobic interaction with Ile10, in all the cis and trans complexes. The carboxyl group of Asp145 in CDK2 and amide group of Asn144 in CDK5 are reported to constitute a salt-bridge with the side chain amino group of Lys33 [16]. In both of our simulated cis-OH bound CDK complexes, this salt-bridge was persistent throughout the simulations (Fig. S3). However, the dynamics was very different in the trans-OH bound CDK5 complex and the salt-bridge went completely missing. Moreover, the terminal hydroxyl group of cis-OH was found to locate very close to the backbone NH of Asp145/Asn144 and form persistent H-bonds. In CDK5, this –OH group also interacted with Lys33 side chain, strengthening the hydrogen bonding network. However, the hydroxyl group of trans-OH was unable to make favourable interactions in either CDK2 or CDK5 during the entire span of simulations. Fig. S4 shows the time evolution of this interaction of cis−/trans-OH inhibitor with Asp145/Asn144 in terms of their distances. The cyclobutyl ring of the inhibitors is involved in CH-π interactions with the benzene ring of Phe80 [39]. In trans-OH-CDK complexes, the CH-π interactions were found to be weaker with ring-ring distances acquiring larger values due to the trans conformation of the polar –OH group (Table S2).

**Figure 4:**
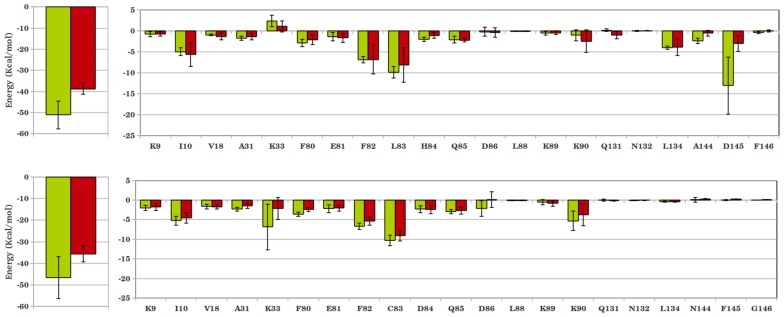
Interaction energies between CDKs and cis/trans-OH inhibitors. (A) CDK2 bound with cis-OH (green) and trans-OH (red); and (B) similar CDK5 complexes. Residue-level decomposition of the total energy is also included, where the significantly contributing residues are noted.

The binding of inhibitors to CDKs was further amplified by calculating their average interaction energies over the final 10 ns simulation trajectory. The total interaction energy of cis-OH was found to be much greater than trans-OH in both CDK2 and CDK5 complexes (Fig. 4). Individual interactions of the protein residues with inhibitor moieties can explain such a difference. For example, the hinge region residues Leu83 in CDK2 and Cys83 in CDK5 interact stronger with imidazole ring of cis-OH than that of the trans-OH inhibitor. Adjacent residues H84 in CDK2 and F82, D86 and K89 in CDK5 also show larger interaction energies with cis-OH. The diminished hydrophobic interaction of trans-OH with F80 is also reflected in the lower interaction energy values. For CDK2-inhibitor complex, the most significant difference in energy was observed due to Asp145, which lay deep inside the substrate binding pocket (−13.08 kcal/mol in cis-OH vs. −3.01 kcal/mol in trans-OH). The neighbouring A144 also displayed considerable lowering in interaction with trans-OH. Leu83 also contributes differently by about 2 kcal/mol in the two complexes (−9.91 kcal/mol in cis- versus −8.13 kcal/mol in trans-OH). The interaction of hydrophobic Phe80 is also found to be more favourable with cis-OH. The contribution of polar Lys33 is repulsive for both the inhibitors, while bound to CDK2. In case of CDK5, however, Lys33 involves in favourable interactions with both the inhibitors. But, it interacts very differently with cis- and trans-OH (−6.88 kcal/mol in cis- and −2.13 kcal/mol in trans-OH) and contributes most significantly toward the difference in total interaction energy in CDK5. Residue Asn144, the analogue of Asp145 in CDK2, contributes negligibly toward inhibitor binding in CDK5. The residues Phe80, Glu81, Phe82 and Cys83 located in the hinge region also showed increased interaction energy with cis-OH. In brief, the analysis suggests that the interaction of cis-OH inhibitor is stronger than trans-OH in both CDK2 and CDK5 and the main contribution toward inhibitor binding comes from Asp145 in CDK2 and Lys33 in CDK5. Time evolutions of the interaction distances also show that the dynamics of these systems differ significantly and the interactions persist longer for cis-OH than the trans-OH inhibitor (Fig. S4, S5).

To get a quantitative comparison of the binding strengths, we computed the free energy of binding of the inhibitors to CDK2 and CDK5 from the simulation-generated trajectories *via* MMPBSA method. Table 2 lists the binding free energies of cis-OH and trans-OH, complexed with active CDKs. The binding of cis-OH was found to be stronger in both CDK2/cyclin E and CDK5/p25 complexes and irrespective of the method of calculation. The computed ΔΔG_binding_ are in very good agreement with experimental data [21].

**Table 2:** Free energy of binding of cis– and trans-OH inhibitors to CDKs from MMPBSA calculations.

Complex	ΔG	ΔΔG_cis-trans_	ΔΔG_cis-trans_ (expt)
cis-OH-CDK2	−20.21±1.05		
trans-OH-CDK2	−18.26±1.43	−1.95	−1.46
cis-OH-CDK5	−20.97±2.6		
trans-OH-CDK5	−19.63±1.67	−1.34	−1.45

All energy values are in kcal/mol and ΔΔG_cis-trans_ = ΔG_cis_−ΔG_trans_.

### Binding of cis-N-acetyl to Active CDK2 and CDK5

The N-acetyl analogue of cis-OH, cis-N-acetyl has shown a ten-fold improved potency over cis-OH against CDK5/p25 *in vitro* (IC_50_ values: 9 vs. 93 nM; Table 1). Moreover, it showed a seven-fold better selectivity for CDK5 over CDK2 (IC_50_ values: 9 vs. 63 nM). To understand these differences, we carried out comparative studies of cis-OH and cis-N-acetyl bound active CDK2 and CDK5 complexes. The N-acetyl bound CDK complexes were simulated for 50 ns and the stability were assured from the convergence of energy components and RMSDs from the crystal structures (data not shown). The comparison of local fluctuation of the protein residues implies a stronger protein-inhibitor interaction in cis-N-acetyl bound CDKs, particularly in CDK5 complex (Fig. S6,S7).

**Figure 5:**
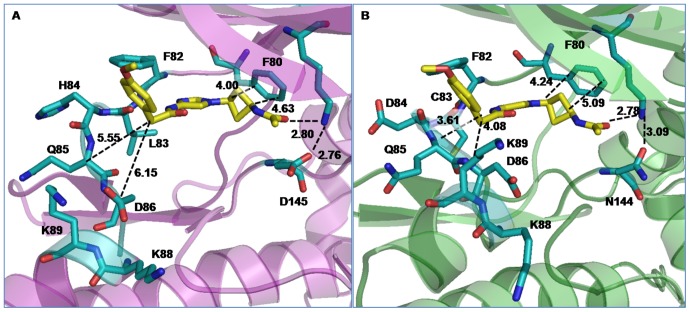
Average structures of the cis-N-acetyl bound CDK complexes. For clarity, only the inhibitors and the adjacent protein residues are shown: (A) cis-N-acetyl bound CDK2, (B) cis-N-acetyl bound CDK5. Possible modes of interactions are indicated by dotted lines with average distances shown. Color scheme is similar to Fig. 3.

To obtain a better understanding of improved potency and selectivity of cis-N-acetyl inhibitor against CDK5/p25 complex, we compared the average structures of the inhibitor bound CDK complexes. This is shown in Fig. 5. For clarity, only the inhibitors and the adjacent protein residues that involve in direct interactions are shown. Most of the interactions present in cis-OH-CDK complexes were seen to be retained in N-acetyl bound CDKs. This includes the interaction of inhibitor imidazole ring with residues Phe82, Leu83/Cys83, His84/Asp84 and the interaction of phenylacetamide moiety with Ile10. The hydrophobic interaction between the inhibitor cyclobutyl ring and Phe80 was also found to persist, in spite of increased ring-ring distances. We observed a bifurcated H-bonding interaction of Lys33:NZ with acetyl oxygen of inhibitor and carbonyl oxygen of Asp145/Asn144 in both CDK2 and CDK5. Such interactions still could maintain the Lys33-Asp145/Asn144 salt-bridge, while providing greater stability to the inhibitor. Although the Lys33-inhibitor interaction was present in cis-OH-CDK5 complex, it has become more persistent in cis-N-acetyl-CDK5 complex due to proximity and larger polarity on the inhibitor (Fig. S8). Other pocket lining residues, *e.g.,* H84/D84, Q85 and D86 also show similar or better binding capacity with N-acetyl inhibitor in CDK5 complex (as exemplified by shorter distances in Fig. 5). Not only the neighbouring pocket residues, analysis further suggests the involvement of certain allosteric residues, such as Lys89 in αD helix - the side chain of which twisted inward to protrude into the binding pocket, thus strengthening the N-acetyl-CDK5 interactions (Fig. S9).

**Figure 6:**
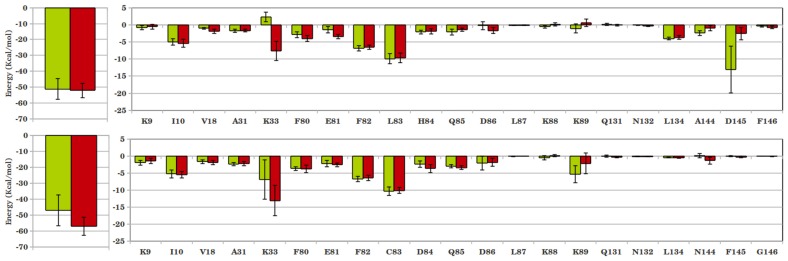
Interaction energies between CDKs and cis-OH/cis-N-acetyl inhibitors. (A) CDK2 bound with cis-OH (green) and cis-N-acetyl (red); (B) similar CDK5 complexes. Residue-level decomposition of the total energy is also included.

**Figure 7:**
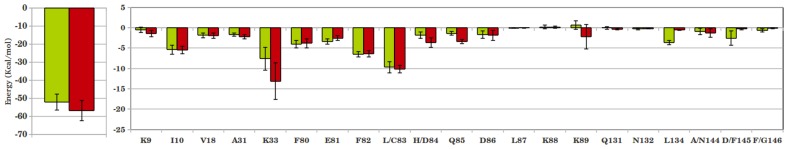
Comparison of the interaction energies between CDK2-cis-N-acetyl (green) and CDK5-cis-N-acetyl (red) complexes. Residue-level decomposition of the total energy is also included.

To quantify the interactions, the inhibitor-protein interaction energies are calculated and shown in Figs. 6 and 7. A marginal increase in total interaction was observed for N-acetyl-CDK2 complex compared to the corresponding cis-OH complex (−52.08 kcal/mol vs. −51.11 kcal/mol). Residue-level analysis shows a marked decrease in interaction of N-acetyl inhibitor with Asp145, which contributed the most in case of cis-OH inhibitor. The adjacent Ala144 also shows a weaker interaction with N-acetyl inhibitor. However, the repulsive interaction of Lys33 with cis-OH reverts to a favourable interaction with cis-N-acetyl, as shown in Fig. 6a. This along with slightly more favourable interactions of Ile10 and hinge region residues Phe80, Glu81 etc. makes cis-N-acetyl as equally potent as cis-OH in inhibiting CDK2. These interactions seem to persist over the entire production phase of the simulations, as shown in the time evolution of a few representative interaction distances (Fig. S10). The cis-N-acetyl bound CDK5 complex, however, shows a large increase in interaction energy by about 10 kcal/mol, compared to the corresponding cis-OH complex (Fig. 6b). Residue-level analysis shows that Lys33 makes almost half of the total difference in energy. The allosteric residue, Lys89 also appears to contribute significantly in the energy difference. Even the hinge region residues, particularly Asp84 and Gln85 contributed more favourably toward the interaction with N-acetyl inhibitor. As Fig. 7 shows, the better selectivity of N-acetyl inhibitor for CDK5 over CDK2 mainly stems from more favourable Lys33 interaction. Additionally, the variant residues Cys83, Asp84 and neighbouring Gln85 help better inhibitor interaction in CDK5. Another variant Asn144 also appears to help inhibitor-CDK5 interactions. Importantly, the interaction of allosteric Lys89 becomes favourable in CDK5 (Fig. S9). In a nutshell, the interaction of residue Lys33 with acetyl group plays the major role in improved potency of cis-N-acetyl inhibitor over cis-OH. The selectivity of cis-N-acetyl for CDK5 presumably comes from the variant residues Cys83, Asp84, Asn144, which modulate the interaction network by subtly restructuring the binding pocket, as a result of which residues Lys33, Lys89 etc. involve in stronger interactions.

To get a better estimate of the binding strengths, we computed the free energy of binding of cis-N-acetyl to CDK2 and CDK5 from the simulation-generated trajectories *via* MMPBSA method (Table 3). The binding energy values go parallel with the higher potency of cis-N-acetyl inhibitor over cis-OH against CDK5/p25, even though these two inhibitors do not show much difference against CDK2/cyclin E complex. The ΔΔG_Nacetyl-OH_ was −2.0 kcal/mol and −0.31 kcal/mol for CDK5 and CDK2, which match favourably with the experimental data. The selectivity of N-acetyl inhibitor for CDK5 complex is also evident from the table, where ΔΔG_CDK5-CDK2_ was computed to be −2.45 kcal/mol from MMPBSA calculation.

**Table 3:** Free energy of binding of cis-OH and cis-N-acetyl inhibitors to CDKs from MMPBSA calculations.

Complex	ΔG	ΔΔG_Nacetyl-OH_	ΔΔG_Nacetyl-OH_ (expt)
cis-OH-CDK2	−20.21±1.05		
cis-N-acetyl-CDK2	−20.52±1.07	−0.31	−0.03
cis-OH-CDK5	−20.97±2.6		
cis-N-acetyl-CDK5	−22.97±3.00	−2.00	−1.41

All energy values are in kcal/mol and ΔΔG_Nacetyl-OH_ = ΔG_Nacetyl_−ΔG_OH_.

### Effect of Mutations

**Figure 8:**
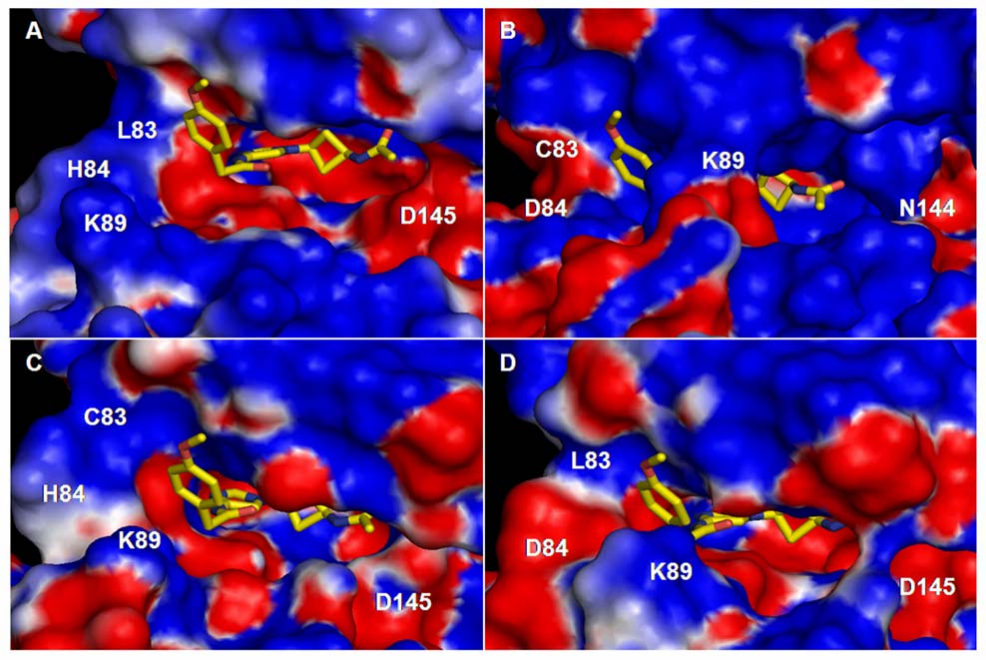
Electrostatic potential maps the substrate binding pocket of CDKs. Potential maps are generated for cis-N-acetyl bound (A) CDK2 (B) CDK5 (C) CDK2:L83C mutant, and (D) CDK2:H84D mutant. Red and blue represent electronegative and electropositive potentials, respectively. The inhibitor is also shown.

To elucidate the physical characteristics of the binding pocket, we have also calculated the solvent accessible surface area (SASA) of the pocket (Table 4, Fig. S11) and mapped its electrostatic potential (Fig. 8). SASA is calculated using naccess program [40] and the average SASA values in Table 4 are obtained from its time evolution in Fig. S11. The electrostatic potential map is obtained from the average structures of the cis-N-acetyl bound CDK complexes using DelPhi program [41]. The calculated SASA values indicate that the binding pocket of CDK5 is smaller than CDK2. The electrostatic potential map shows that the pocket is more electropositive in CDK5 complex, particularly deep inside the cavity. This is due to the Asp145/Asn144 variant and inward movement of allosteric Lys89 (see Fig. S8). Recall that the N-acetyl group of the inhibitor contains many electronegative atoms, which thus find a suitable environment to remain stable. This can also explain why cis-OH with a smaller electronegative –OH headgroup binds relatively weakly to the pocket than N-acetyl.

**Table 4:** Average solvent accessible surface area (SASA) of the substrate binding pocket of CDKs.

Protein complex	SASA (Å^2^)	Std. dev.
CDK2 wild type	5240.20	92.63
CDK5 wild type	4754.80	170.74
CDK2:L83C variant	5149.64	85.81
CDK2:H84D variant	4876.07	97.42

SASA is calculated by removing the cis-N-acetyl inhibitor from the pocket and rolling a probe of radius 1.4 Å across the pocket.

To check if the other two CDK2 variants contribute to pocket volume, even though they reside exterior to the binding pocket, we created the mutants, CDK2:L83C and CDK2:H84D. These complexes were also simulated for 50 ns after equilibration. The computed volumes and electrostatic potential map of these mutants are also included in Table 4 and Fig. 8. As evident from the table and potential map, both mutations reduce the pocket volume and induce similar changes to the electrostatic potential as seen in CDK5 complex. Taken together, the inhibitors bind relatively strongly to CDK5 binding pocket due to the smaller volume and electropositive nature of the binding pocket. The atomic-level details on CDK-inhibitor interactions presented here could help the design of more specific CDK inhibitors.

### Binding of Roscovitine to Active CDK2 and CDK5

**Figure 9:**
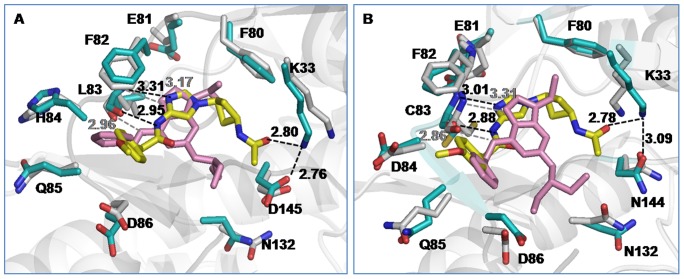
Superimposed structures of cis-N-acetyl and roscovitine bound CDK complexes: (A) CDK2 (B) CDK5. In roscovitine-CDK complexes, the drug and protein residues are shown in pink and grey, respectively. Remaining color scheme is similar to Fig. 3.

The binding of N-acetyl inhibitor to CDKs is also compared with the binding of commercially available CDK inhibitor, roscovitine [42]. As table 1 indicates, the inhibitory effect of N-acetyl on active CDK2 and CDK5 is much greater than roscovitine. To understand this differential inhibition, a comparative analysis of their mode of binding to CDKs has been carried out from the 20 ns simulation trajectory of each roscovitine-bound complex. Fig. 9 presents the time-averaged structures of N-acetyl and roscovitine bound CDK complexes, superimposed on each other. Clearly, the peripheral moieties of both N-acetyl and roscovitine make similar contacts with CDKs. For example, Leu83/Cys83 interact with imidazole ring of N-acetyl and purine ring of roscovitine with equal strength, as exemplified by their similar H-bonding distances in Fig. 9. The terminal phenyl moiety involves in hydrophobic interaction with Ile10 in both inhibitor bound complexes. However, the characteristic interactions of N-acetyl with Lys33 and Asp145/Asn144 were completely missing for roscovitine (Fig. 9). The time evolution of such an interaction distance between Lys33 and the closest inhibitor atom shows that roscovitine could never reach to the base of the deep binding cavity of CDKs (Fig. S12). Moreover, the stacking interaction of cyclobutyl ring with Phe80 was also absent in roscovitine bound CDK complexes.

**Figure 10:**
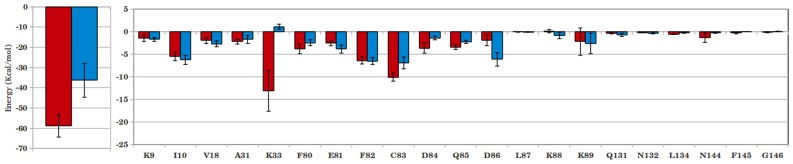
Interaction energy of CDK5 with cis-N-acetyl (red) and roscovitine (blue). Residue-level decomposition of the total energy is also included.

The calculation of residue-level interaction energies reflects a similar trend (Fig. 10). Even though a few neighbouring residues, such as Ile10, Val18, Glu81 and Asp86 have similar or marginally higher interaction with roscovitine, most of the other pocket residues contribute more toward N-acetyl interaction. Major contributor toward the larger binding strength of N-acetyl was Lys33, followed by hinge region residues Leu83/Cys83, His84/Asp84, Gln85. The hydrophobic Phe80 and the CDK2/CDK5 variant residue Asp145/Asn144 also contribute more favourably toward the N-acetyl inhibitor. Consequently, the total interaction energy of N-acetyl with CDKs turns out to be much greater than roscovitine. The decomposition of total energy into electrostatic and van der Waal components indicates that N-acetyl fared over roscovitine through the electrostatic interaction (Table 5). The six fold increase of electrostatic component for the former mainly stems from the polar interaction of its N-acetyl group with Lys33, Asp145/Asn144, which reside deep into the CDK binding pocket. Hence, the future strategy for designing more potent and specific CDK inhibitors might incorporate polar functional groups that can reach deep into the CDK binding pocket through a hydrophobic linker, such as the cyclobutyl ring here.

**Table 5:** The contribution of electrostatic and van der Waals energy toward the total interactions in inhibitor-CDK5 complexes.

Complex	Total Energy	Electrostatic	van der Waals
cis-N-acetyl-CDK5	−53.53±5.56	−27.5±6.12	−26.03±2.17
Roscovitine-CDK5	−36.28±8.36	−6.12±2.11	−31.86±1.5

All energies are in kcal/mol.

### Conclusions

Cis-substituted cyclobutyl-4-aminoimidazole inhibitors have been identified as novel CDK5 inhibitors that gave improved enzyme and cellular potency with many fold selectivity over CDK2. The molecular basis of higher potency and selectivity of this class of inhibitors over commercially available drugs is also unknown. Here we present atomic-level details of the interactions of some of these CDK-inhibitor complexes to understand these differences. Results suggest that the aminoimidazole inhibitors can reach deep into the substrate-binding pocket through the linker cyclobutyl group. Moreover, they involve in strong electrostatic interactions with CDK residues Lys33, Asp145/Asn144 that reside at the base of the cavity. The better selectivity of these inhibitors for CDK5 mainly stems from the variant residues Cys83, Asp84, Asn144, which modulate the interaction network by subtly restructuring the binding pocket and realigning the allosteric residues, Lys33, Lys89. This turns the CDK5 pocket more electropositive and smaller in volume for more favourable interactions with molecules carrying multiple electronegative sites.

The results are validated by comparing the computed free energy of binding of the imidazole inhibitors to CDKs with the available experimental values. Moreover, the mode of binding of the commercially available drug, roscovitine to CDKs in the simulated complexes is also compared to the available crystal structure. An excellent match has been observed in both instances, which tempted us to conclude that the future strategy for designing more potent and specific CDK inhibitors could involve the incorporation of polar functional groups at the tip of the inhibitor molecules, which can go deep into the binding pocket through a hydrophobic linker.

## Supporting Information

Figure S1 The C_α_ root mean squared deviations (RMSD) of CDKs bound to cis- and trans-OH inhibitors. Time evolution is shown for final 35 ns for cis-OH-CDK2 (black), trans-OH-CDK2 (red), cis-OH-CDK5 (green), and trans-OH-CDK5 (blue) complexes.
(TIF)

Figure S2 RMSDs of the inhibitors bound to CDKs. Black: cis-OH bound to CDK2, red: trans-OH bound to CDK2, green: cis-OH bound to CDK5, blue: trans-OH bound to CDK5.
(TIF)

Figure S3 The time evolution of the salt-bridge between Asp145/Asn144 and Lys33 in CDKs. Results are shown for the distances (A) between carboxyl group of Asp145 and the side chain amino group of Lys33 in CDK2 and (B) between amide group of Asn144 and the side chain amino group of Lys33 in CDK5. Color scheme: Red for cis-OH bound and black for trans-OH bound CDK complex. See Fig. 3 for atom notations.
(TIF)

Figure S4 Time evolution of the interaction of cis−/trans-OH inhibitor with (A) Asp145 in CDK2 and (B) Asn144 in CDK5. Interactions are shown in terms of the distance between the hydroxyl group of the inhibitors and the backbone NH of Asp145/Asn144. Color scheme is similar to Fig. S3. See Fig. 3 for atom notations.
(TIF)

Figure S5 Time evolution of the interaction of cis- and trans-OH inhibitors with Lys33 in CDK5. Interactions are shown in terms of the distance between the hydroxyl group of the inhibitors and the side chain N of Lys33. Color scheme is similar to Fig. S3. See Fig. 3 for atom notations.
(TIF)

Figure S6 Comparison of local fluctuations of (A) CDK2 and (B) CDK5 residues bound to cis-OH (black) and cis-N-acetyl (red) inhibitors.
(TIF)

Figure S7 Comparison of local fluctuations of CDK2 (black) and CDK5 (red) residues bound to cis-N-acetyl inhibitor.
(TIF)

Figure S8 Time evolution of the interaction of cis-OH (black) and cis-N-acetyl (red) inhibitors with Lys33 in CDK5. Interactions are shown in terms of the distances between the side chain N of Lys33 and hydroxyl group of cis-OH and nitrogen of N-acetyl, respectively. See Figs. 3 and 5 for atom notations.
(TIF)

Figure S9 Orientations of residues around N-acetyl inhibitor in (A) CDK2 (B) CDK5 (C) CDK2:L83C variant, and (D) CDK2:H84D variant. Figure clearly shows the intrusion of residue K89 into the CDK5 binding pocket in panel (B). A similar change of orientation of K89 is also seen in the variant CDK2:H84D (panel D). Color scheme is similar to Fig. 3.
(TIF)

Figure S10 Time evolution of the interaction of cis-OH (black) and cis-N-acetyl (red) inhibitors with (A) Asp145 and (B) Lys33 in CDK2. Interactions are shown in terms of the distance between the hydroxyl group of cis-OH and nitrogen of N-acetyl with the backbone NH of Asp145 and the side chain N of Lys33, respectively. See Figs. 3 and 5 for atom notations.
(TIF)

Figure S11 Time evolution of the solvent accessible surface area of the binding pocket of CDK2 (black), CDK5 (red), CDK2:L83C mutant (green), and CDK2:H84D mutant (blue).
(TIF)

Figure S12 Time evolution of the interaction of roscovitine (black) and cis-N-acetyl (red) inhibitor with Lys33 in (A) CDK2 and (B) CDK5. Interactions are shown in terms of the distances between the side chain N of Lys33 and closest roscovitine atom and nitrogen of N-acetyl, respectively.
(TIF)

Table S1 List of systems studied.
(DOC)

Table S2 Average distance and energy between cyclobutyl ring of inhibitor and phenyl ring of CDK:Phe80. For distance calculations, centre of masses are considered.
(DOC)

File S1 Full reference 27.
(DOC)
