# Supplementary figures and images for: Molecular Basis of Differential Selectivity of Cyclobutyl-Substituted Imidazole Inhibitors against CDKs: Insights for Rational Drug Design
Source: PLoS One. 2013 Sep 13;8(9):e73836. doi: 10.1371/journal.pone.0073836 (PMC3772847; doi:10.1371/journal.pone.0073836)

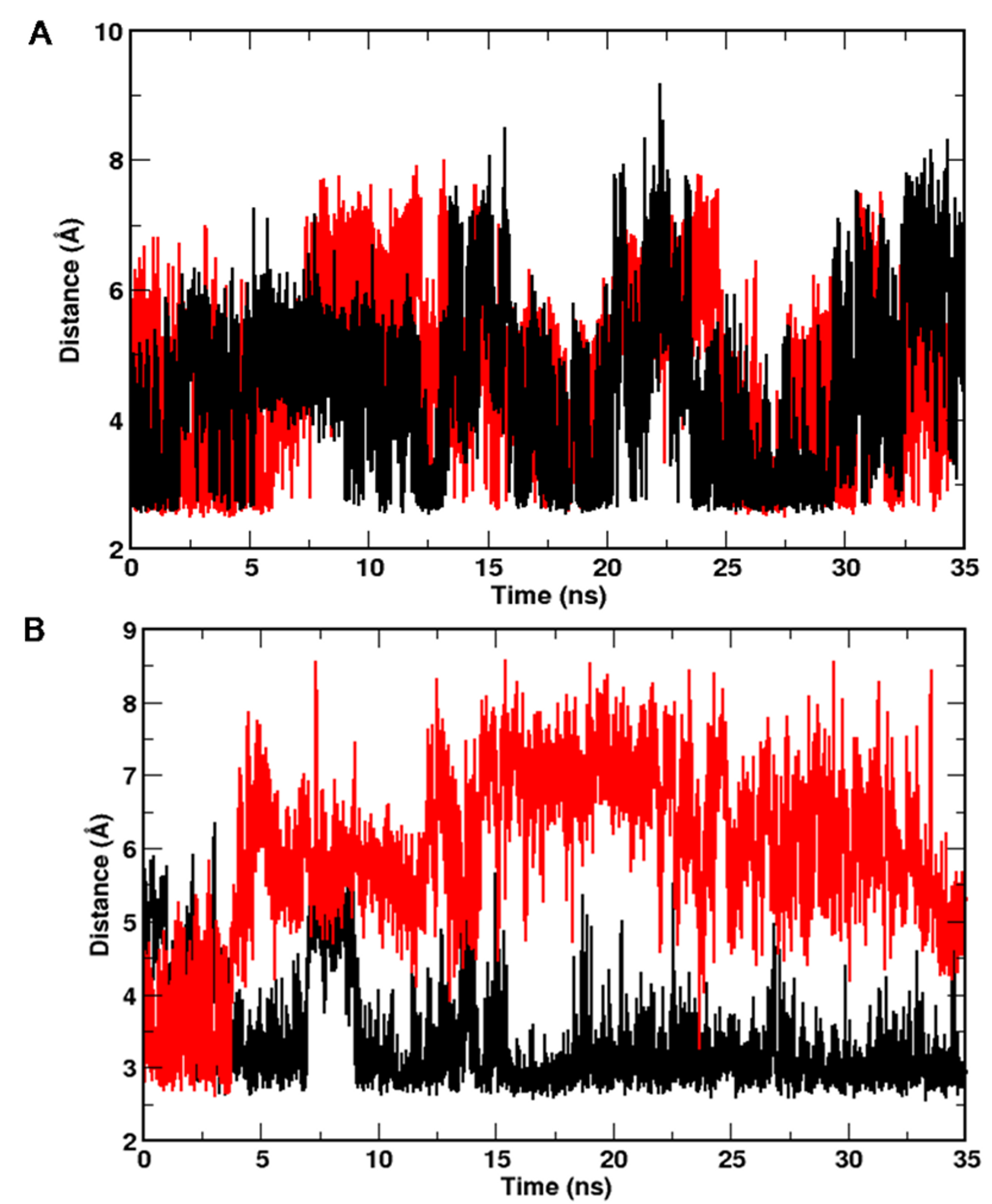

Supplement: Figure S3 — The time evolution of the salt-bridge between Asp145/Asn144 and Lys33 in CDKs. Results are shown for the distances (A) between carboxyl group of Asp145 and the side chain amino group of Lys33 in CDK2 and (B) between amide group of Asn144 and the side chain amino group of Lys33 in CDK5. Color scheme: Red for cis-OH bound and black for trans-OH bound CDK complex. See Fig. 3 for atom notations. (TIF) [file pone.0073836.s003.tif]

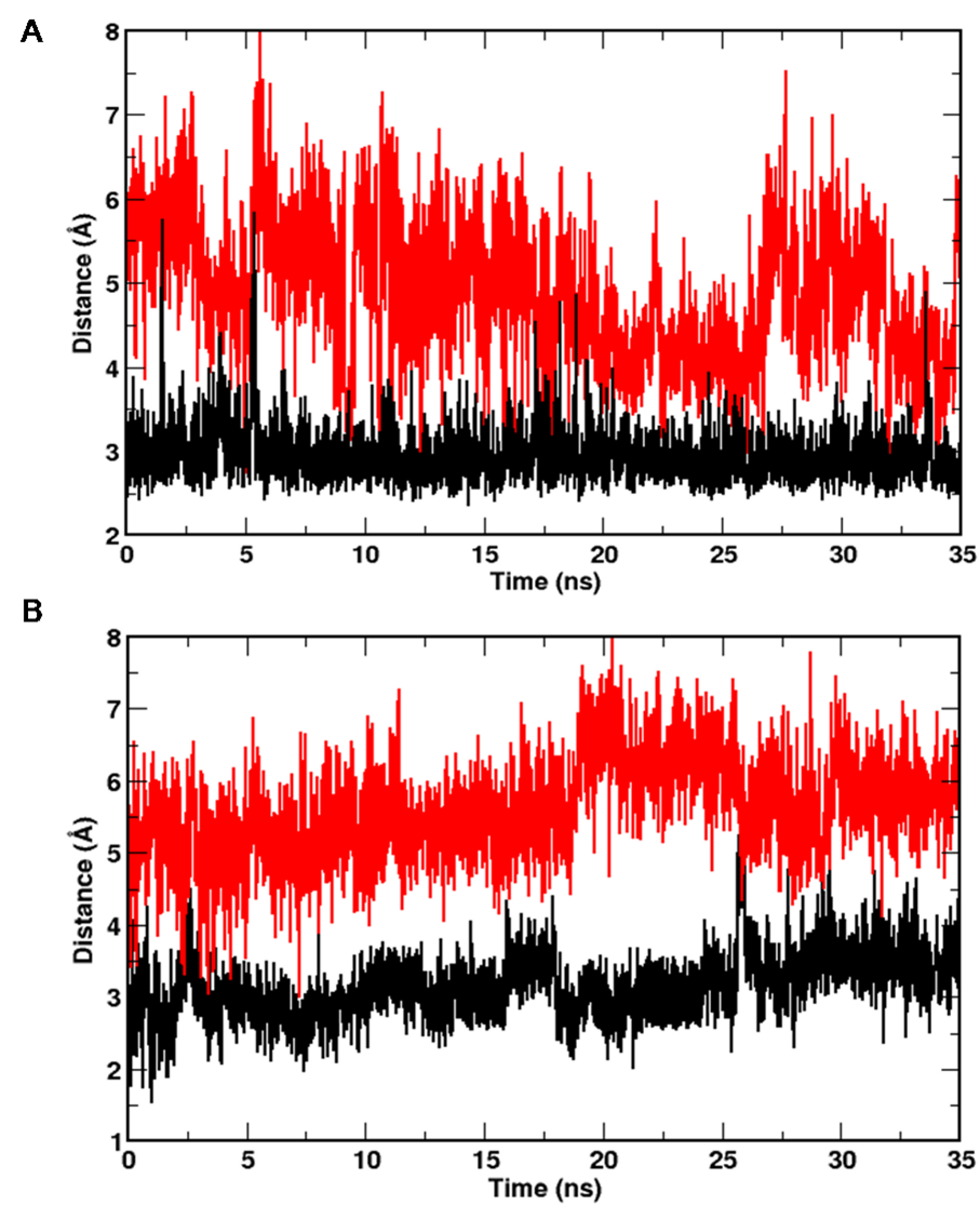

Supplement: Figure S4 — Time evolution of the interaction of cis−/trans-OH inhibitor with (A) Asp145 in CDK2 and (B) Asn144 in CDK5. Interactions are shown in terms of the distance between the hydroxyl group of the inhibitors and the backbone NH of Asp145/Asn144. Color scheme is similar to Fig. S3. See Fig. 3 for atom notations. (TIF) [file pone.0073836.s004.tif]

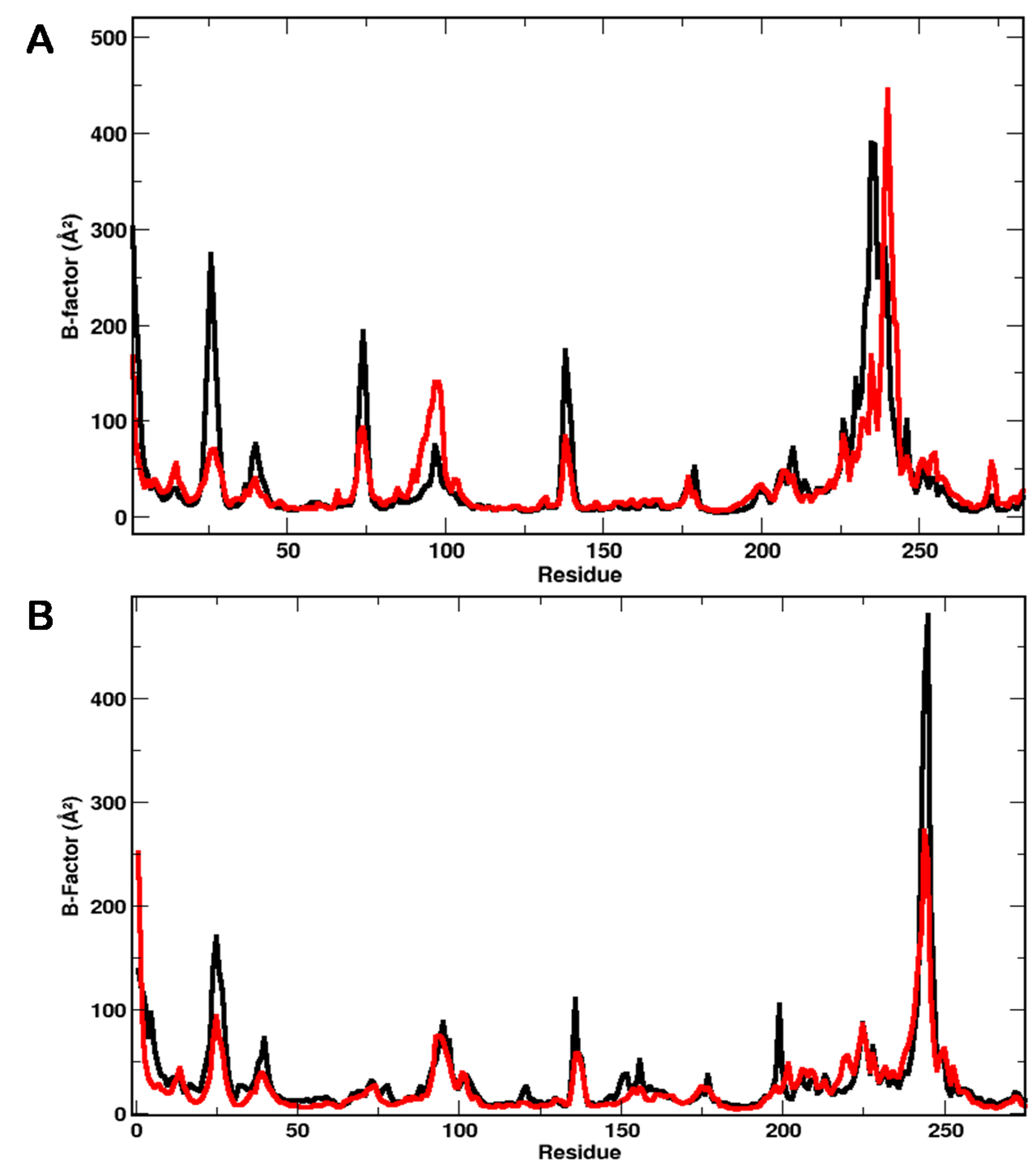

Supplement: Figure S6 — Comparison of local fluctuations of (A) CDK2 and (B) CDK5 residues bound to cis-OH (black) and cis-N-acetyl (red) inhibitors. (TIF) [file pone.0073836.s006.tif]

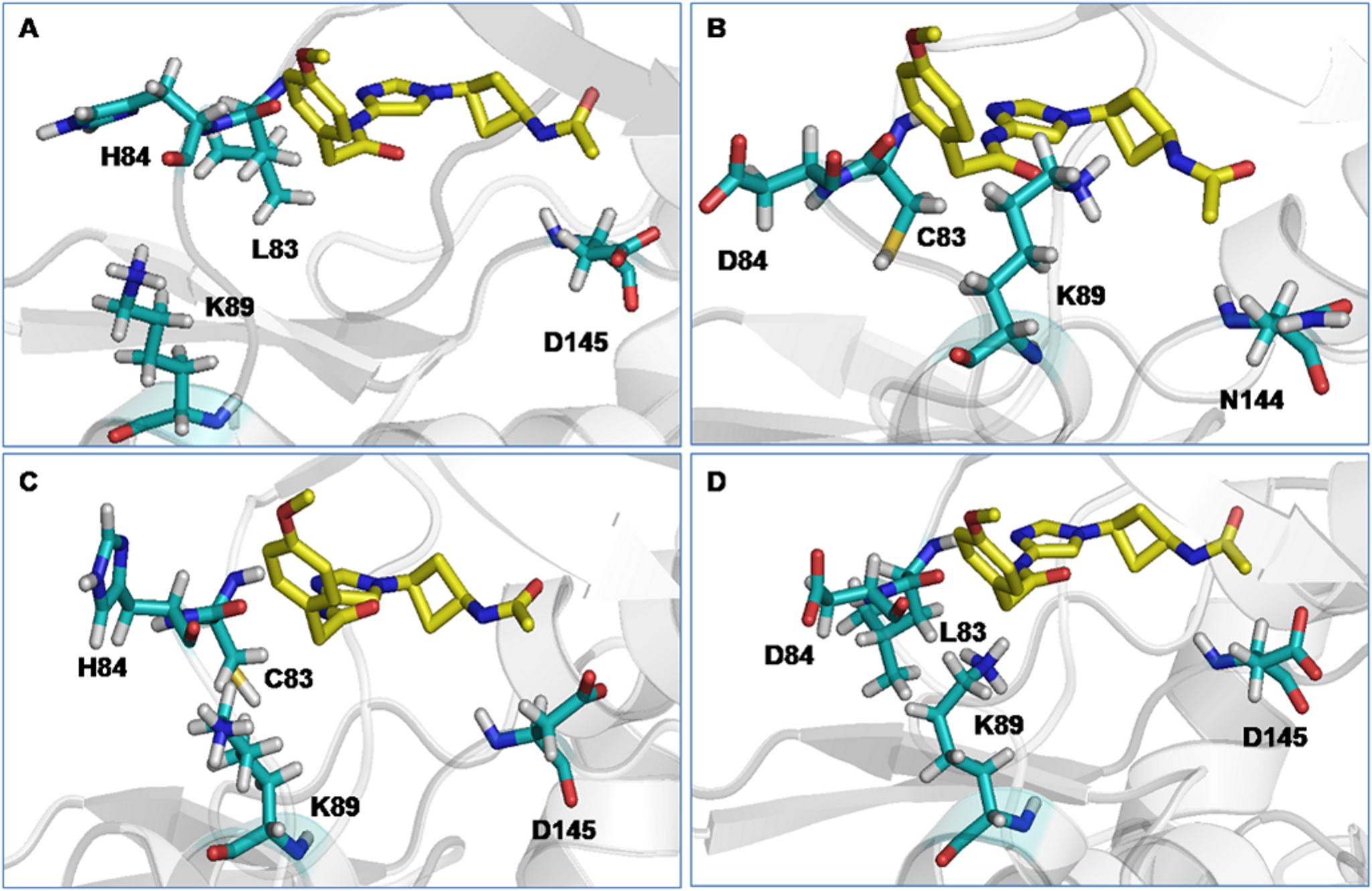

Supplement: Figure S9 — Orientations of residues around N-acetyl inhibitor in (A) CDK2 (B) CDK5 (C) CDK2:L83C variant, and (D) CDK2:H84D variant. Figure clearly shows the intrusion of residue K89 into the CDK5 binding pocket in panel (B). A similar change of orientation of K89 is also seen in the variant CDK2:H84D (panel D). Color scheme is similar to Fig. 3. (TIF) [file pone.0073836.s009.tif]

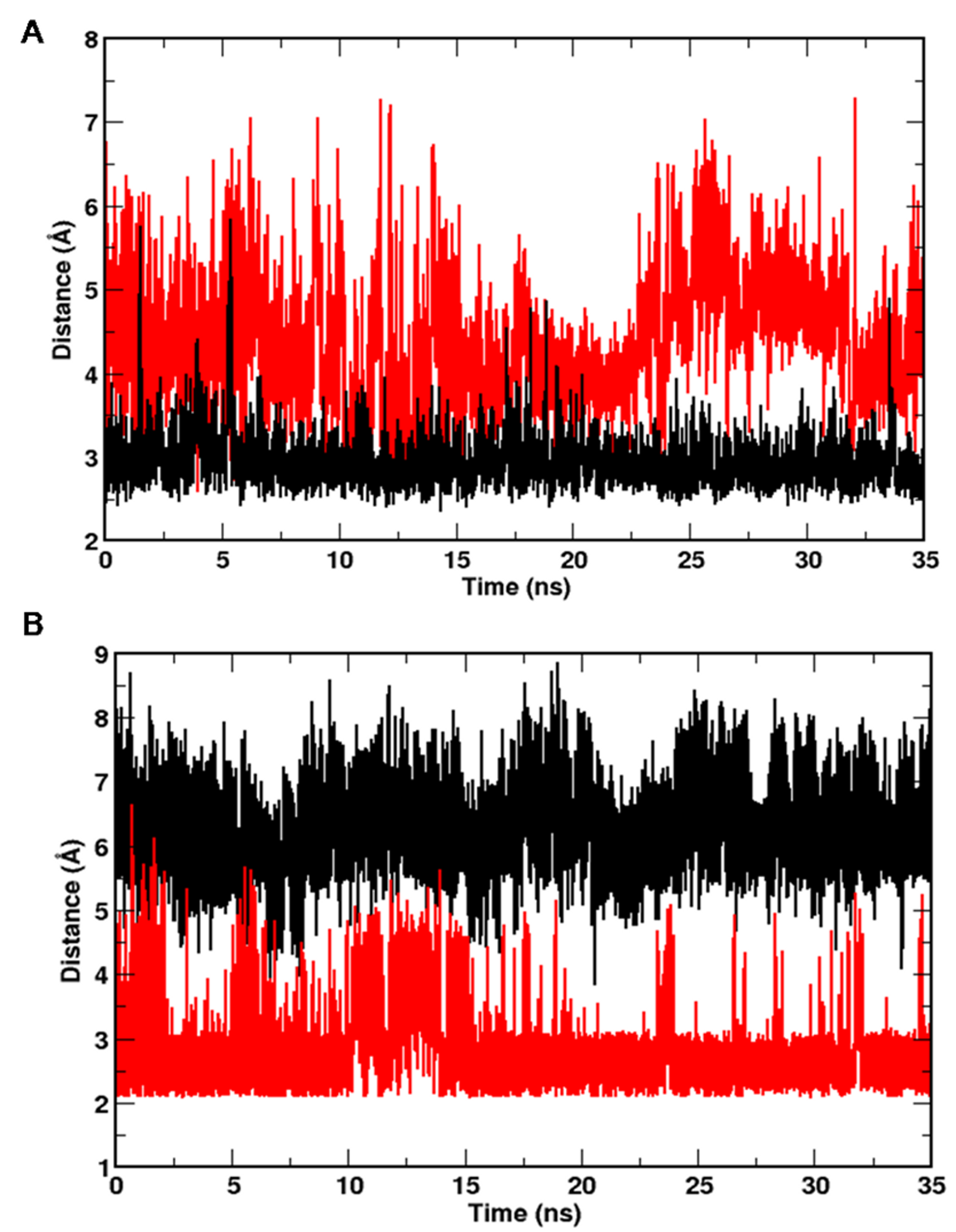

Supplement: Figure S10 — Time evolution of the interaction of cis-OH (black) and cis-N-acetyl (red) inhibitors with (A) Asp145 and (B) Lys33 in CDK2. Interactions are shown in terms of the distance between the hydroxyl group of cis-OH and nitrogen of N-acetyl with the backbone NH of Asp145 and the side chain N of Lys33, respectively. See Figs. 3 and 5 for atom notations. (TIF) [file pone.0073836.s010.tif]

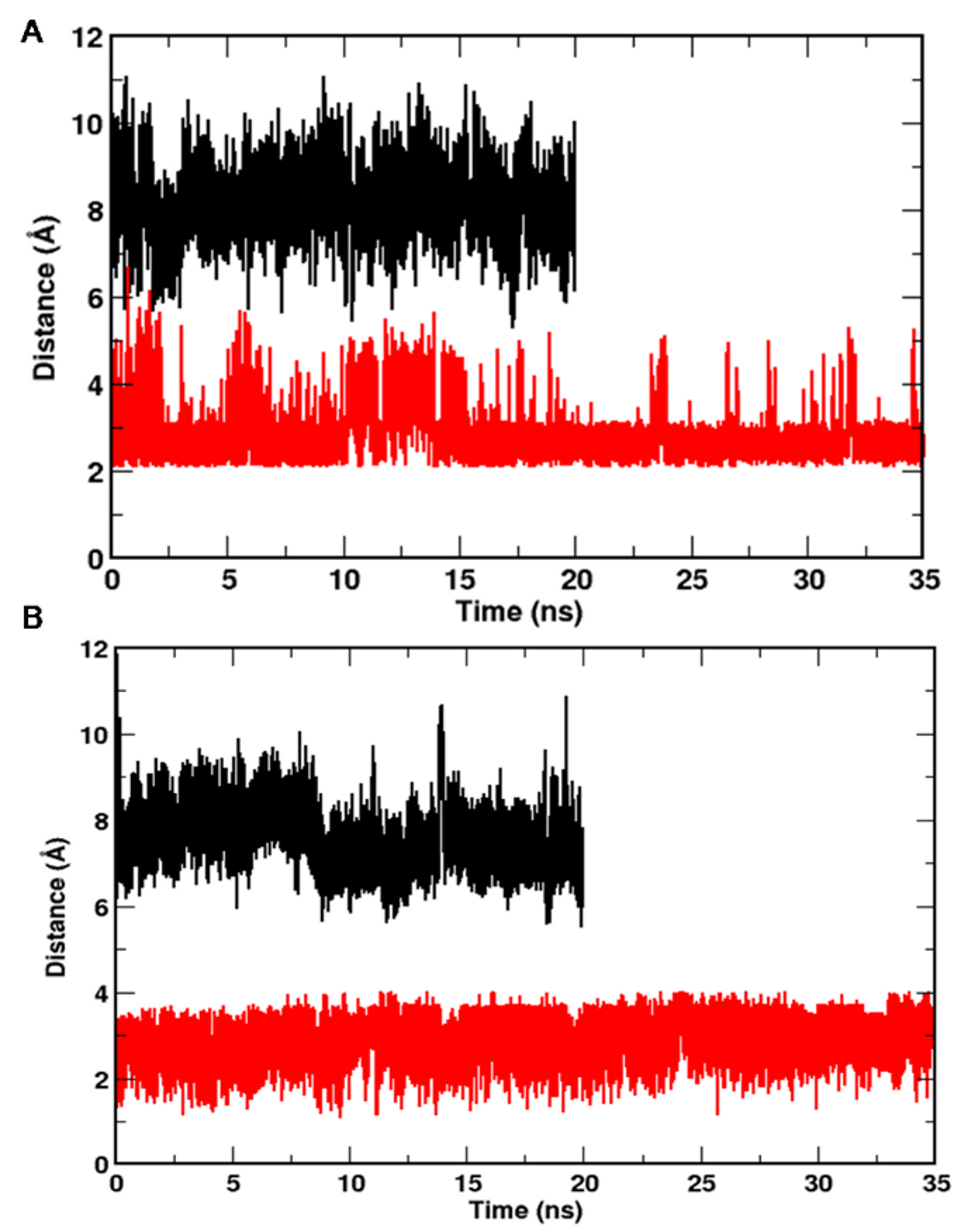

Supplement: Figure S12 — Time evolution of the interaction of roscovitine (black) and cis-N-acetyl (red) inhibitor with Lys33 in (A) CDK2 and (B) CDK5. Interactions are shown in terms of the distances between the side chain N of Lys33 and closest roscovitine atom and nitrogen of N-acetyl, respectively. (TIF) [file pone.0073836.s012.tif]
