# Supplementary material for: Molecular Basis of Differential Selectivity of Cyclobutyl-Substituted Imidazole Inhibitors against CDKs: Insights for Rational Drug Design
Source: PLoS One. 2013 Sep 13;8(9):e73836. doi: 10.1371/journal.pone.0073836 (PMC3772847; doi:10.1371/journal.pone.0073836)
Supplement: Table S1 — List of systems studied. (DOC) [file pone.0073836.s013.doc]

**Table S1.** List of systems studied.

| System | Protein-inhibitor Complex | Duration of Simulation |
| --- | --- | --- |
| 1 | CDK2/CyclinE/cis-OH | 50 ns |
| 2 | CDK2/CyclinE/trans-OH | 50 ns |
| 3 | CDK5/p25/cis-OH | 50 ns |
| 4 | CDK5/p25/trans-OH | 50 ns |
| 5 | CDK2/CycilnE/cis-N-acetyl | 50 ns |
| 6 | CDK5/p25/cis-N-acetyl | 50 ns |
| 7 | CDK2:L83C/CyclinE/cis-N-acetyl | 50 ns |
| 8 | CDK2:H84D/CyclinE/cis-N-acetyl | 50 ns |
| 9 | CDK2/CyclinE/roscovitine | 20 ns |
| 10 | CDK5/p25/roscovitine | 20 ns |
