# Supplementary material for: Molecular Basis of Differential Selectivity of Cyclobutyl-Substituted Imidazole Inhibitors against CDKs: Insights for Rational Drug Design
Source: PLoS One. 2013 Sep 13;8(9):e73836. doi: 10.1371/journal.pone.0073836 (PMC3772847; doi:10.1371/journal.pone.0073836)
Supplement: Table S2 — Average distance and energy between cyclobutyl ring of inhibitor and phenyl ring of CDK:Phe80. For distance calculations, centre of masses are considered. (DOC) [file pone.0073836.s014.doc]

**Table S2.** Average distance and energy between cyclobutyl ring of inhibitor and phenyl ring of CDK: Phe80. For distance calculations, centre of masses are considered.

| Complex | Distance (Å) | Energy (kcal/mol) |
| --- | --- | --- |
| Cis-OH-CDK2 | 4.23±0.11 | -3.99±0.3 |
| Trans-OH-CDK2 | 4.75±0.43 | -3.17±0.85 |
| Cis-OH-CDK5 | 4.5±0.1 | -3.6±0.5 |
| Trans-OH-CDK5 | 5.05±0.5 | -2.2±1.15 |
