# Supplementary material for: Molecular Basis of Differential Selectivity of Cyclobutyl-Substituted Imidazole Inhibitors against CDKs: Insights for Rational Drug Design
Source: PLoS One. 2013 Sep 13;8(9):e73836. doi: 10.1371/journal.pone.0073836 (PMC3772847; doi:10.1371/journal.pone.0073836)
Supplement: File S1 — Full reference 27. (DOC) [file pone.0073836.s015.doc]

Full reference 27.

27. Gaussian 03, Revision E.01, Frisch MJ, Trucks GW, Schlegel HB, Scuseria GE, Robb MA, Cheeseman JR, Montgomery JA, Vreven JrT, Kudin KN, Burant JC, Millam JM, Iyengar SS, Tomasi J, Barone V, Mennucci B, Cossi M, Scalmani G, Rega N, Petersson GA, Nakatsuji H, Hada M, Ehara M, Toyota K, Fukuda R, Hasegawa J, Ishida M, Nakajima T, Honda Y, Kitao O, Nakai H, Klene M, Li X, Knox JE, Hratchian HP, Cross JB, Bakken V, Adamo C, Jaramillo J, Gomperts R, Stratmann RE, Yazyev O, Austin AJ, Cammi R, Pomelli C, Ochterski JW, Ayala PY, Morokuma K, Voth GA, Salvador P, Dannenberg JJ, Zakrzewski VG, Dapprich S, Daniels AD, Strain MC, Farkas O, Malick DK, Rabuck AD, Raghavachari K, Foresman JB, Ortiz JV, Cui Q, Baboul AG, Clifford S, Cioslowski J, Stefanov BB, Liu G, Liashenko A, Piskorz P, Komaromi I, Martin RL, Fox DJ, Keith T, Al-Laham MA, Peng CY, Nanayakkara A, Challacombe M, Gill PMW, Johnson B,Chen W, Wong MW, Gonzalez C, and Pople JA, Gaussian, Inc., Wallingford CT, 2004.
